# An overview of osteoporosis and frailty in the elderly

**DOI:** 10.1186/s12891-017-1403-x

**Published:** 2017-01-26

**Authors:** Guowei Li, Lehana Thabane, Alexandra Papaioannou, George Ioannidis, Mitchell A. H. Levine, Jonathan D. Adachi

**Affiliations:** 1 Department of Clinical Epidemiology & Biostatistics, McMaster University, 1280 Main Street West, Hamilton, ON L8S 4L8 Canada; 2 St. Joseph’s Healthcare Hamilton, McMaster University, 25 Charlton Avenue East, Hamilton, ON L8N 1Y2 Canada; 3 Programs for Assessment of Technology in Health, Centre for Evaluation of Medicines, Hamilton, ON L8N 1Y3 Canada; 4 Department of Medicine, McMaster University, 1280 Main Street West, Hamilton, ON L8S 4L8 Canada; 5 St. Joseph’s Healthcare Hamilton, McMaster University, 25 Charlton Avenue East, Hamilton, ON L8N 1Y2 Canada

**Keywords:** Osteoporosis, Osteoporotic fractures, Frailty, Geriatrics, Ageing

## Abstract

Osteoporosis and osteoporotic fractures remain significant public health challenges worldwide. Recently the concept of frailty in relation to osteoporosis in the elderly has been increasingly accepted, with emerging studies measuring frailty as a predictor of osteoporotic fractures. In this overview, we reviewed the relationship between frailty and osteoporosis, described the approaches to measuring the grades of frailty, and presented current studies and future research directions investigating osteoporosis and frailty in the elderly. It is concluded that measuring the grades of frailty in the elderly could assist in the assessment, management and decision-making for osteoporosis and osteoporotic fractures at a clinical research level and at a health care policy level.

## Background

Osteoporosis is defined as a systemic skeletal disease with the characteristics of low bone mass and microarchitectural deterioration of bone tissues [1]. In clinical practice, osteoporosis is usually diagnosed by the bone mineral density (BMD) criteria or the occurrence of a fragility fracture. Based on the BMD criteria, osteoporosis is diagnosed by a BMD of 2.5 standard deviations or more below the mean of a young healthy adult women reference population (T-score ≤ −2.5) [2]. Osteoporosis results in increased bone fragility and subsequent accumulated fracture risk. With decreased BMD as people age, osteoporosis becomes more prevalent among older individuals [3]. As the population ages worldwide, the number of osteoporotic fractures is growing substantially. In western countries, the lifetime risk of any osteoporotic fracture remains very high, lying within the range of 40–50% for women and 13–22% for men [4]. For the year 2000, it was estimated that 9 million new osteoporotic fractures occurred globally, of which 1.6 million were hip fractures and 1.4 million were clinical vertebral fractures [5]. In the US, there are more than 2 million fractures annually attributed to osteoporosis, including 550,000 vertebral fractures and 300,000 hip fractures [6, 7]. Osteoporotic fractures in the elderly are usually followed by hospitalization, long-term care, impaired quality of life, disability and death [8]. Therefore osteoporosis and osteoporotic fractures remain significant public health challenges worldwide.

Recently, the concept of frailty in relation to osteoporosis in the elderly has been increasingly accepted, with emerging studies measuring frailty as a predictor of osteoporotic fractures [9]. Frailty is defined as a dynamic clinical condition with increased vulnerability which results from aging-related degeneration across psychological, physical and social functioning [10, 11]. Frailty is accelerating in the aging population, with an overall prevalence of 10.7% in community-dwellers aged ≥ 65 years worldwide [12]. Moreover, it is estimated that 25–50% of older adults aged ≥ 85 years are frail [9]. Frailty is mainly caused by the complex aging mechanisms that are determined by underlying genetic, epigenetic and environmental factors [9]. However, other multifactorial elements such as sarcopenia, inflammation, malnutrition, co-morbidities, hormonal insufficiency, etc., can also result in frailty in the elderly [13–15]. The fundamental of the relationship between frailty and osteoporosis relies on the fact that, the frailer an individual is, the greater the likelihood that the individual will have a prevalent osteoporotic fracture and the higher the risk of a fracture in the future [16, 17]. Quantifying the degree of frailty could aid in the assessment, management and decision-making for the elderly at a clinical research level and at a health care policy level [9].

### Measuring grades of frailty in assessing risk of osteoporotic fractures

A frailty instrument should be multidimensional, able to capture the grades of frailty, and qualified to serve as a screening or evaluation tool [18]. At present, two predominant approaches are being widely used in measuring the degree of frailty in the elderly: the phenotype model [19] and the frailty index of deficit accumulation [20]. Table 1 lists the respective components of the phenotype model and the frailty index of deficit accumulation. The phenotype model is calculated from five physical indicators (exhaustion, low physical activity, weakness, slow walking and unintentional weight loss) [19]. Each indicator is scored as either 0 or 1 and therefore the total score ranges from 0 to 5 points. The phenotype model categorizes the elderly into robust, pre-frail or frail groups by the cut-points of the total score of 0, 1–2 and ≥ 3 points respectively [19]. By contrast, the frailty index chooses a variety of individual health deficits to measure the cumulative effect and quantify the degree of frailty [20]. Generally, the deficits cover the domains of symptoms and signs, comorbidities, activities of daily living, and social relations and social support [21, 22]. Though each individual deficit may not carry an imminent threat of adverse health outcomes, the deficit accumulation contributes to the increased risk [23]. The frailty index approach does not require the same deficits or the same number of variables to build a frailty index [24]. Previous studies have selected 30 to 70 health deficits in creating a frailty index [25]. However it has been recommended to include at least 30 to 40 deficits in total to construct a frailty index [22]. Each deficit is dichotomized or polychotomized and mapped on an interval scale between 0 and 1, in order to reflect the frequency or severity of the deficit [22]. Subsequently, the frailty index is calculated by summing up all the deficit values and dividing by the whole number of the deficits included. For example, if a frailty index includes 35 deficits, and an individual has 6 deficits with each scored as 1 point (6 point total), 2 deficits with each scored as 0.5 (1 point total), and the remaining 27 deficits with each scored of 0, then the frailty index would be 7 divided by 35 giving an index of 0.2.

**Table 1:** Components of the phenotype model and the frailty index of deficit accumulation

Approach to measuring grades of frailty	Components
The phenotype model^a^	Exhaustion
	Low physical activity
	Weakness
	Slow walking
	Unintentional weight loss
The frailty index of deficit accumulation^b^	Deficits of symptoms and signs
	Comorbidities
	Deficits of activities of daily living
	Deficits of social relations and social support

^a^he phenotype model is based on five physical indicators

^b^The frailty index of deficit accumulation is calculated from a variety of individual health deficits

Evidence has shown that both the phenotype model and the frailty index are predictive of osteoporotic fractures independent of chronological age in the elderly [26–29]. For instance, the Study of Osteoporotic Fractures (SOF) assessed the relationship between a phenotype model and risk of fractures in 6724 women aged ≥ 69 years with a mean follow-up of 9 years [26]. They reported higher hip fracture risk (hazard ratio (HR) = 1.40, 95% confidence interval (CI): 1.03–1.90) and non-spine fracture risk (HR = 1.25, 95% CI: 1.05–1.49) in frail women, compared with their robust peers. In addition, one study using data from the Canadian Multicentre Osteoporosis Study (CaMos) constructed a 30-item frailty index in 9423 adults with a mean age of 62 years and a 10-year follow-up [29]. Results indicated a significant HR of 1.18 for hip fractures and 1.30 for clinical vertebral fractures for every 0.10 increase in the frailty index.

In quantifying the risk of adverse health outcomes, even with statistical overlap and convergence, some studies argued that the continuous frailty index of deficit accumulation showed higher discriminatory ability than the categorical phenotype model [9, 30–32]. However, other comparative studies have found that the phenotype model was comparable with the frailty index in predicting risk of adverse outcomes [33–35]. For instance, results from a Chinese study presented similar predictive accuracy of the frailty index and the phenotype model for risk of mortality and physical limitation [33]. Likewise, our study using data from the Global Longitudinal Study of Osteoporosis in Women (GLOW) 3-year Hamilton Cohort did not find a significant difference in predictive accuracy for risk of osteoporotic fractures, even though the frailty index approach tended to yield more precise estimates as compared with the phenotype model [35]. These findings may imply the flexibility in the choice of frailty models in population-based settings for the elderly. However, the frailty index is usually considered as a research tool because of the amount of information it requires to complete the assessment, while the phenotype model can be pragmatically applied in geriatric clinical practice [33, 35]. Indeed, it has been suggested that the combined or sequential use of the two approaches should be implemented for the elderly, given that the frailty index and the phenotype model provide complementary and distinct clinical information on the risk profiles [36].

### Current research investigating osteoporosis and frailty in the elderly

There are some studies using derivations of the phenotype model or other categorical frailty models to measure the degree of frailty. Results from the comparisons between them and the original phenotype model have been published, with comparable model performances reported in different populations [3, 34, 37–40]. In addition, evidence indicates that the phenotype model may require model calibration or redevelopment. Some studies have raised concerns about the scoring algorithm for the phenotype model, with emerging reports showing that not all the components contributed equally to the prediction of adverse health outcomes [7, 41–43]. Previous findings have suggested that slow walking appears to be the most important risk factor for adverse outcomes among the five indicators included in the phenotype model [41, 42]. Moreover, low predictive accuracy of the phenotype model in the prediction of adverse outcomes has been reported [35, 37, 44]. For instance, one study found the phenotype model could not differentiate the healthy elderly from those with unplanned hospital admission and falls, with an area under the receiver operating characteristic curve (AUC) value of 0.50 and 0.52 respectively [44]. Similarly, another study reported an AUC value of 0.55 for non-spine fractures and 0.63 for hip fractures in 6701 women using the phenotype model [37].

Regarding the frailty index approach, much of the literature focuses on the comparison between the frailty index and the phenotype model in predicting risk of adverse outcomes [33–35, 38, 45–47]. Of note, it may be methodologically challenging to directly compare the continuous frailty index and the categorical phenotype model. One study based on the GLOW 3-year Hamilton Cohort used three strategies to perform direct comparisons between the frailty index and the phenotype model by (1) investigating the associations between the adverse outcomes and respective per one-fifth (20%) increase of the frailty index and the phenotype model; (2) trichotomizing the frailty index according to the overlap in the density distribution of the frailty index by the robust, pre-frail and frail groups defined by the phenotype model; and (3) trichotomizing the participants based on a predicted probability function of outcomes predicted by the frailty index [35]. All the three strategies yielded comparable predictive accuracy of the frailty index and the phenotype model in predicting risk of adverse outcomes. Additionally, some studies compare the frailty index with other existing tools for predictions of risk of osteoporotic fractures. For instance, there was one study comparing the frailty index with the fracture risk assessment tool (FRAX) in prediction of risk of major osteoporotic fracture (spine, hip, upper arm or shoulder, or wrist) and hip fracture in 3985 elderly women [48]. The frailty index was found to be comparable with FRAX in predicting risk of major osteoporotic fractures and hip fracture, indicating that measuring grades of frailty may aid in fracture risk evaluation and fracture prevention for the elderly. Of note, we observed similar results in the women stratified by taking or not taking anti-osteoporotic treatments and/or supplementation, which indicated that the prediction of frailty index and FRAX in major osteoporotic fractures was not significantly influenced by the effect of anti-osteoporotic treatments and/or supplementation [48]. However, further studies are needed to evaluate whether the assessment of frailty would be a useful addition to FRAX to improve predictive accuracy for risk of fractures in the elderly. Furthermore, despite abundant studies investigating the trajectory nature of the frailty index in the elderly, limited evidence is available for the change of frailty before and after an osteoporotic fracture. In our study, we aimed to assess the change of the frailty index before and after onset of a major osteoporotic fracture during follow-up in the elderly women [46]. We found that the increase of the frailty index was significantly larger in the women who experienced a major osteoporotic fracture than their controls, indicating their greater deficit accumulation and accelerating frailty after a major osteoporotic fracture [46]. Investigating the transition nature by the change of frailty index before and after a major osteoporotic fracture may be useful to serve as an indicator for the effect of treatments or interventions [16]. For example, the change of frailty may be used to identify the minimally important differences (MIDs) in a fracture intervention study, taking into account the frailty transition nature [46]. Though results of the prediction of frailty status in risk of osteoporotic fractures are consistent in the literature, it still remains largely unknown whether frailty is a cause or a consequence of osteoporosis. For instance, some studies have reported no significant cross-sectional relationship between frailty and osteoporosis [49, 50], though frailty and osteoporosis share similar biological pathways and common risk factors such as advanced age, low physical activity, weight loss and cognitive decline [51]. More high-quality evidence is required to further clarify the association between frailty and osteoporosis dependently or independently of the aging process.

### Future research directions

The phenotype model and the frailty index have been shown to be useful tools in predicting risk of osteoporotic fractures in the elderly. Future research may need to justify the validity and reliability of the frailty instruments in clinical settings and research studies, before they can be fully used to guide clinical decision-making [16]. Moreover, measures of frailty need to be tested against the effect of treatments or interventions in studies aiming to prevent or treat frailty. Likewise, the effect of frailty on recovery after a fracture or prevention of a secondary fracture in the elderly warrants further investigation. Besides, given that there is lack of an operational definition for frailty and sarcopenia, it would be a worthwhile endeavor to investigate the combined or sequential use of the instruments (or risk assessment tools) for frailty and sarcopenia in the elderly. Information on assessing frailty and sarcopenia may, together or in parallel of an osteoporosis assessment tool, provide more comprehensive vision of the risks to develop hard clinical outcomes for osteoporotic patients. Other research areas needed to be examined in depth include: 1) the relationship between frailty and osteoporotic fractures in different populations; 2) integration of elements of frailty to FRAX to determine whether higher predictive accuracy can be achieved; and 3) whether interventions in the pre-frail older adults can prevent osteoporotic fractures.

In addition, more studies are warranted to evaluate the role of the frailty instruments as an outcome measure, rather than just a risk assessment tool. As the frailer an elderly is, the greater the risk of osteoporotic fractures, quantifying the degree of frailty may be also helpful as an outcome measure, especially for some short-term fracture intervention studies. Furthermore, understanding the complexity of aging and frailty in the elderly necessitates more exploration of the aging nature *per se*.

## Conclusion

In summary, we have presented an overview of the relationship between osteoporosis and frailty in the elderly. Measuring the degree of frailty in older adults by the frailty index and/or the phenotype model could assist in the assessment, management and decision-making for osteoporosis and osteoporotic fractures at a clinical research level and at a health care policy level. More evidence is needed to examine whether interventions in the pre-frail older adults can prevent osteoporotic fractures and to further support its usefulness and application of the frailty assessment in the elderly with osteoporosis in different populations.

## Abbreviations

AUC: Area under the receiver operating characteristic curve
BMD: Bone mineral density
CaMos: Canadian multicentre osteoporosis study
CI: Confidence interval
FRAX: Fracture risk assessment tool
GLOW: Global longitudinal study of osteoporosis in women
HR: Hazard ratio
MIDs: Minimally important differences
SOF: Study of osteoporotic fractures
